# Blood biomarkers and/or neuropsychological assessment? Optimization of the screening protocols for the early stages of Alzheimer's disease

**DOI:** 10.1002/alz70856_102569

**Published:** 2025-12-25

**Authors:** Cristina Delgado‐Alonso, María Valles‐Salgado, Esther Valiente‐Gordillo, Isabel Ortega‐Madueño, María Cruz Cárdenas‐Fernández, María Díez‐Cirarda, Juan Ignacio López‐Carbonero, Ulises Gómez‐Pinedo, Jorge Matias‐Guiu, María José Gil‐Moreno, Jordi A Matias‐Guiu

**Affiliations:** ^1^ Hospital Clinico San Carlos, Madrid, Madrid, Spain; ^2^ Institute of Laboratory Medicine, IdSSC, Hospital Clínico San Carlos, Madrid, Madrid, Spain; ^3^ Hospital Clínico San Carlos, Madrid, Madrid, Spain; ^4^ Hospital Clinico Universitario San Carlos, Madrid, Madrid, Spain

## Abstract

**Background:**

The detection of early biological and clinical changes in Alzheimer's disease (AD) is mandatory for therapeutic interventions. Blood *p*‐tau markers, such as *p*‐tau181 and *p*‐tau217, have shown promising performance in identifying patients with early amyloid pathology, positioning themselves as potential first‐line diagnostic tests. The development of plasma biomarkers for AD requires understanding their clinical implications and redefining the role of cognitive assessment in screening and diagnosing patients with early symptoms. Our study aimed to determine the optimal combination of biomarkers and cognitive tests to detect AD in its early stages.

**Method:**

We included 124 patients consulting for memory loss with no functional impairment. The mean age was 69.50±6.54 years old, and 73 (58.9%) were women. The mean MMSE was 27.16±3.07. All patients were evaluated with neuropsychological assessment and CSF biomarkers. According to the results of the CSF biomarkers, patients were categorized as AD or non‐AD. Patients with suspicion of other neurodegenerative disorders were not included. Serum *p*‐tau181 and *p*‐tau217 were measured afterwards with Lumipulse G600II. The neuropsychological assessment comprised the following cognitive tests: Mini‐Mental State Examination (MMSE), Addenbrooke's Cognitive Examination III (ACE‐III), digit span, Corsi test, Trail Making Test, Symbol Digit Modalities test, Boston Naming Test, Free and Cued Selective Reminding Test (FCSRT), Rey‐Osterrieth Complex Figure (ROCF), verbal fluency, Visual Object and Space Perception Battery, Judgement Line Orientation test, Stroop Color Word and Interference Test, and Tower of London.

**Result:**

The AUC for *p*‐tau181 and *p*‐tau217 was 0.935 and 0.886, respectively. The best AUC for neuropsychological tests was 0.865 when age, FCSRT, and ROCF‐memory were combined. A logistic regression model including ptau181 and ACE‐III (memory) showed an AUC of 0.949, and the AUC was 0.964 when combining ptau181 and ROCF (memory).

**Conclusion:**

Our study suggests that the combination of neuropsychological tests and blood biomarkers could improve diagnostic performance in the early stages of AD. However, the fact that the biomarker's importance in the statistical models was greater partially challenges the utility of cognitive assessments, particularly for screening purposes and in situations where clinical time may be limited.

